# Supplementary material for: Orbitofrontal PV interneurons modulate social interaction via default mode network dynamics
Source: Commun Biol. 2026 Apr 24;9:573. doi: 10.1038/s42003-026-10060-y (PMC13109428; doi:10.1038/s42003-026-10060-y)
Supplement: Supplementary file 1 — Supplemental material [file 42003_2026_10060_MOESM1_ESM.pdf]

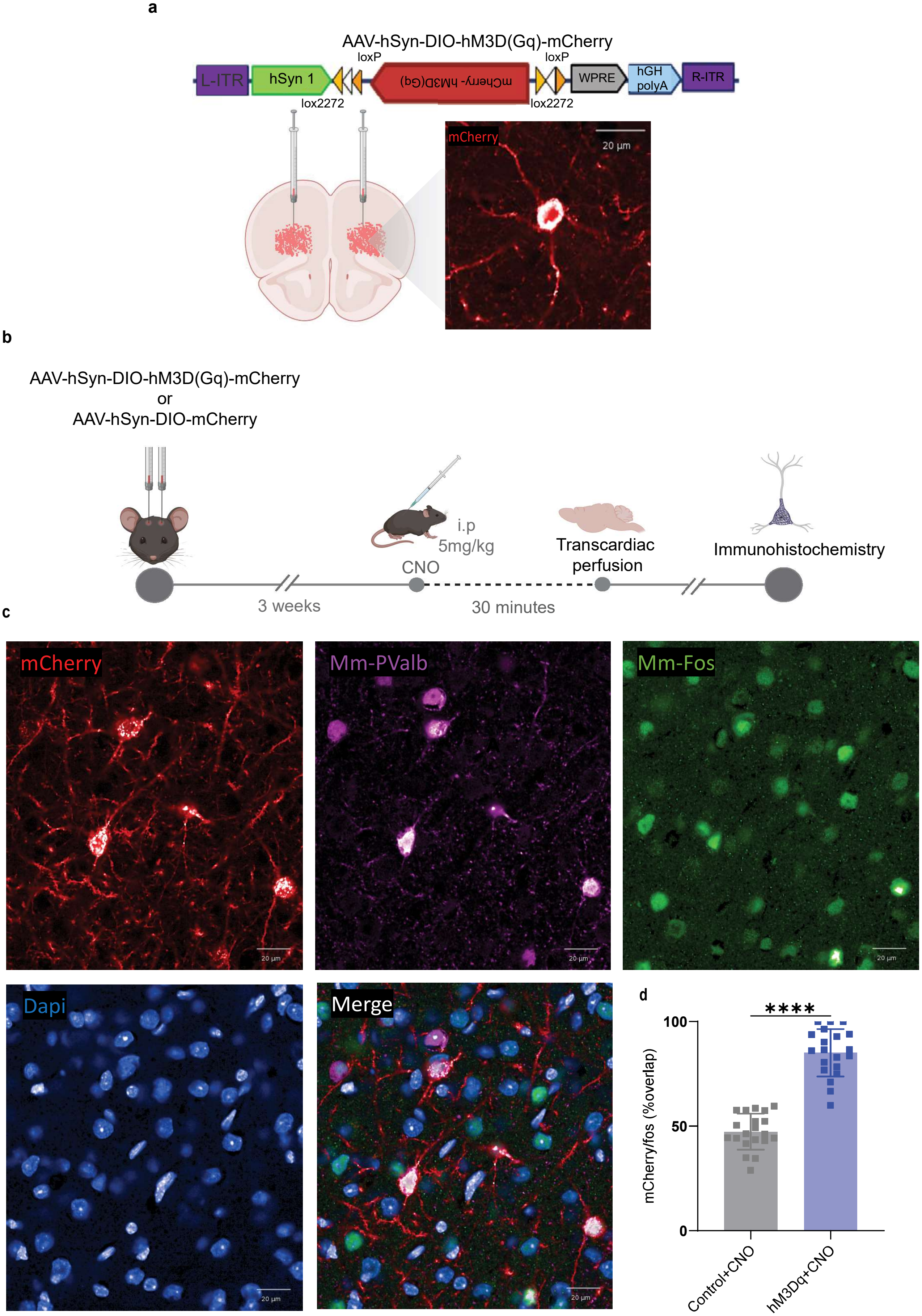

Figure S1. Chemogenetic activation of hM3Dq receptors enhances Fos expression in PV interneurons. **a**, viral injection illustration. **b**, Immunohistochemistry experimental design. **c**, Representative confocal images showing PV, mCherry, and Fos expression, along with DAPI-positive cell nuclei in the OFC. **d**, Bar graph representing the proportion of Fos-positive cells within the population of mCherry-positive cells after CNO injection in hM3Dq (n=20) versus Control (n=20) (unpaired T test, ****p<0.0001). PV interneuron: parvalbumin interneuron. AAV: adeno-associated virus. OFC: orbitofrontal cortex. CNO: clozapine-N-oxide. Error bars represent mean ± standard deviation (SD).

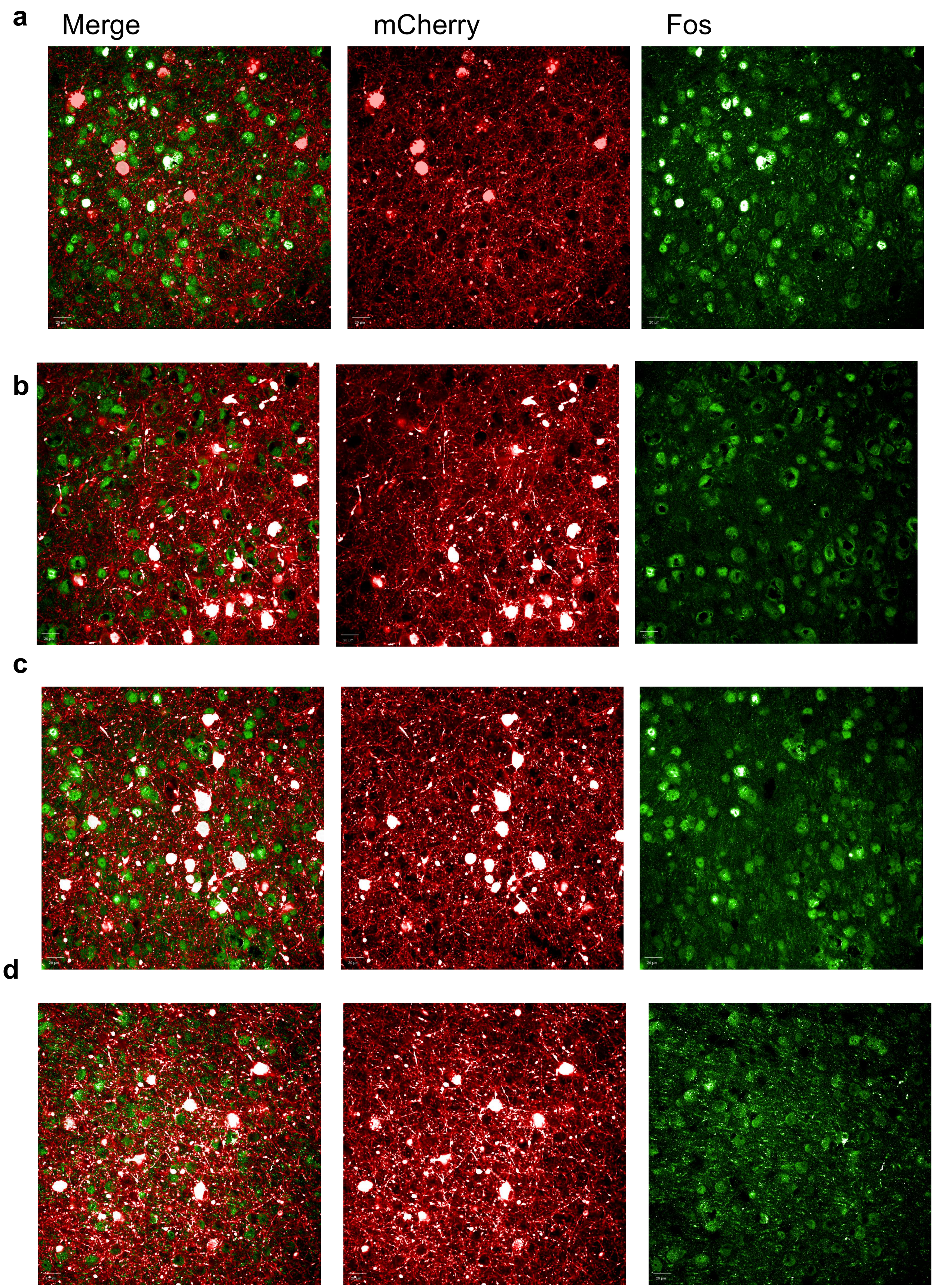

Figure S2.**a-d, Confocal images comparing the proportion of Fos-positive cells in control group.**

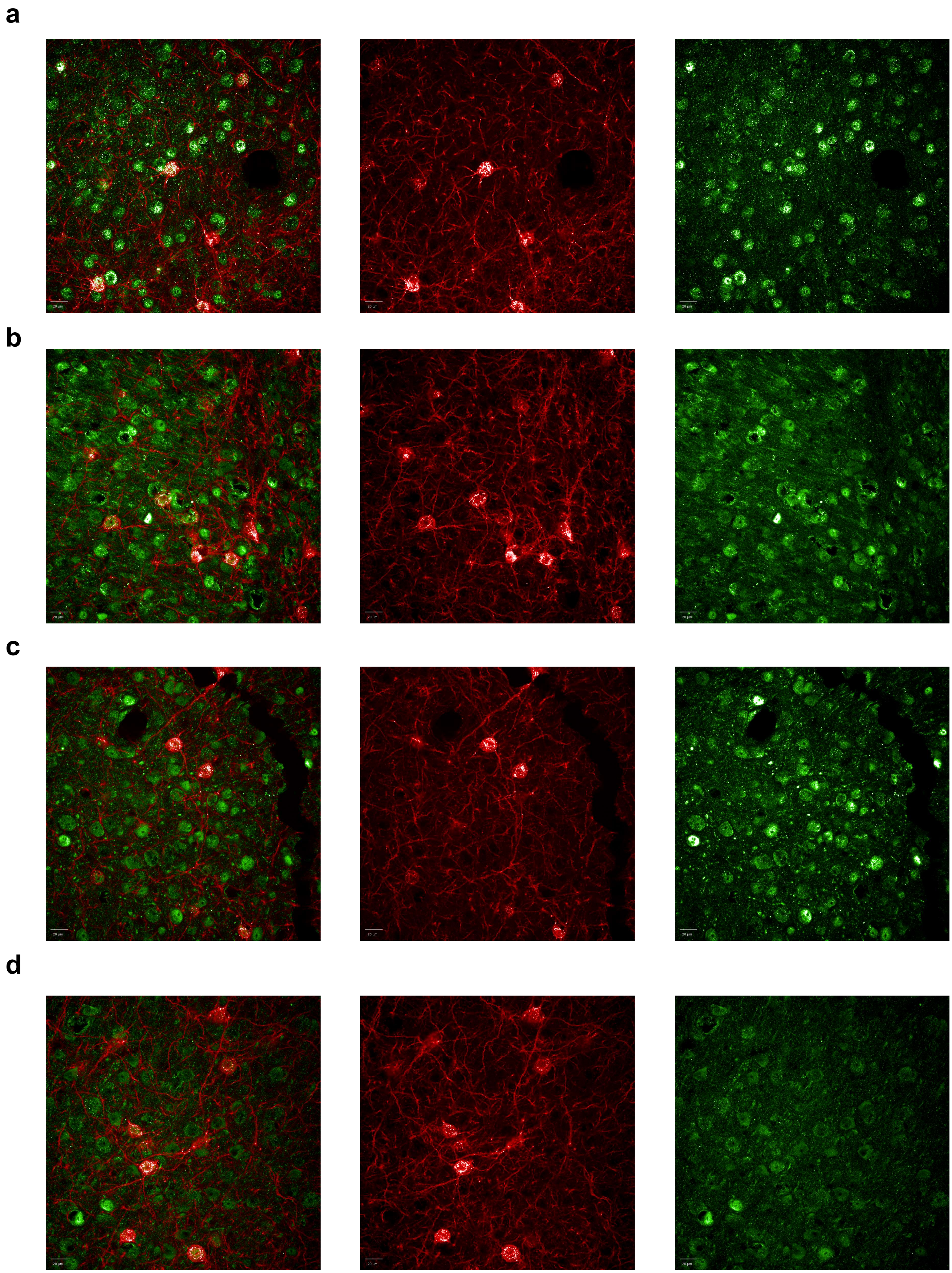

Figure S3.**a-d, Confocal images comparing the proportion of Fos-positive cells in hM3Dq group.**

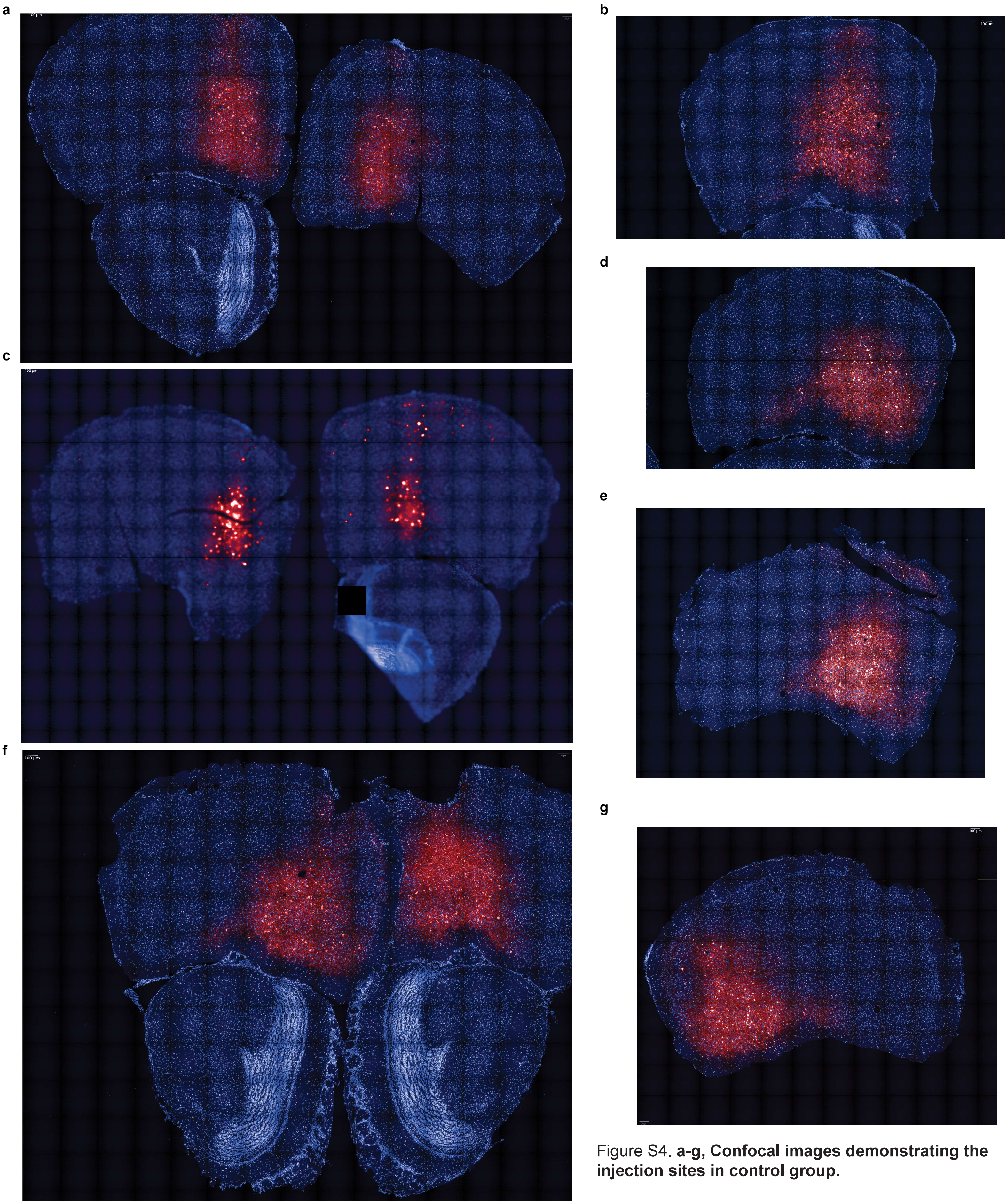

Figure S4. **a-g, Confocal images demonstrating the injection sites in control group.**

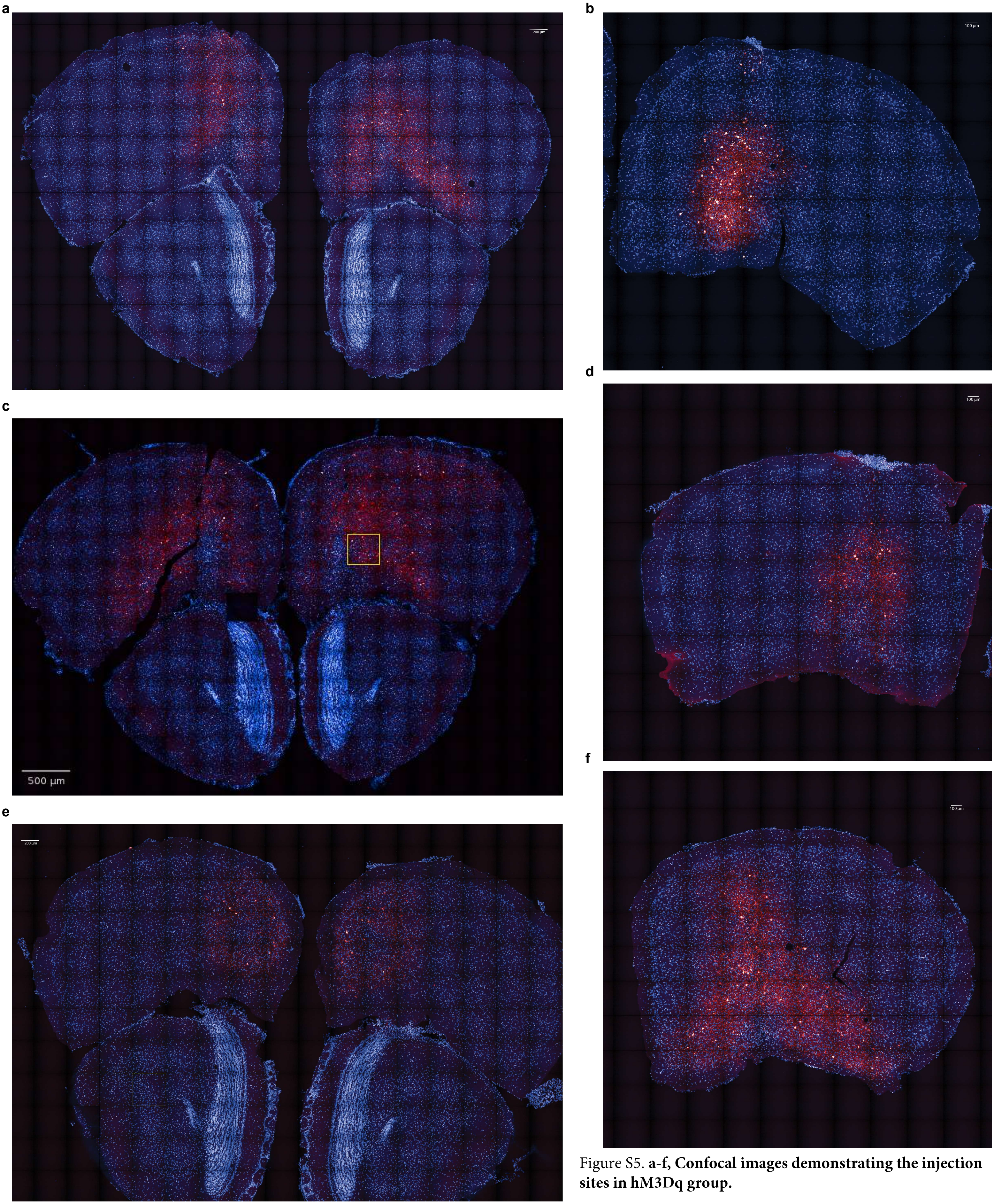

Figure S5. **a-f, Confocal images demonstrating the injection sites in hM3Dq group.**

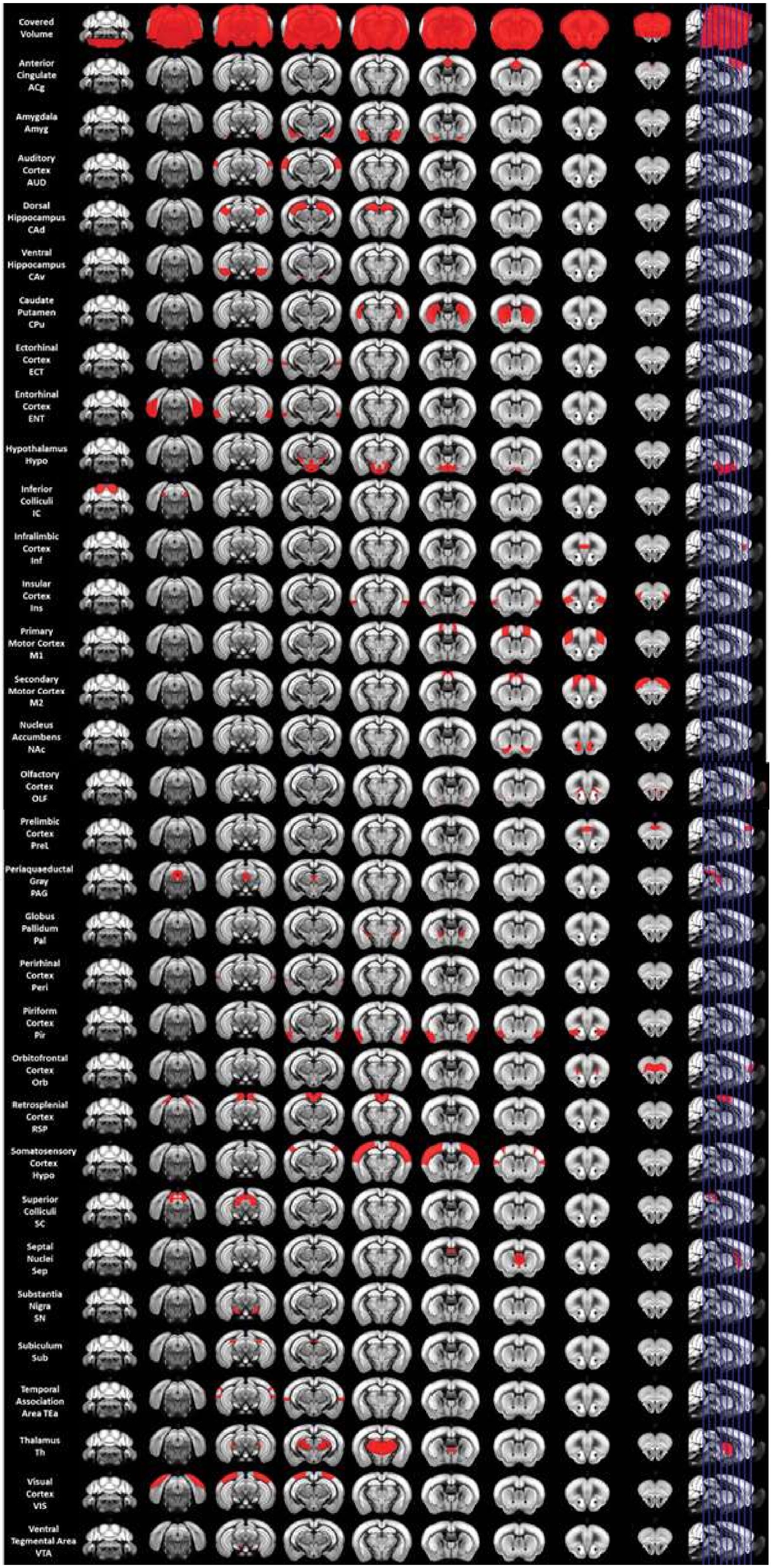

Figure S6**. Illustration of the ROI locations.**

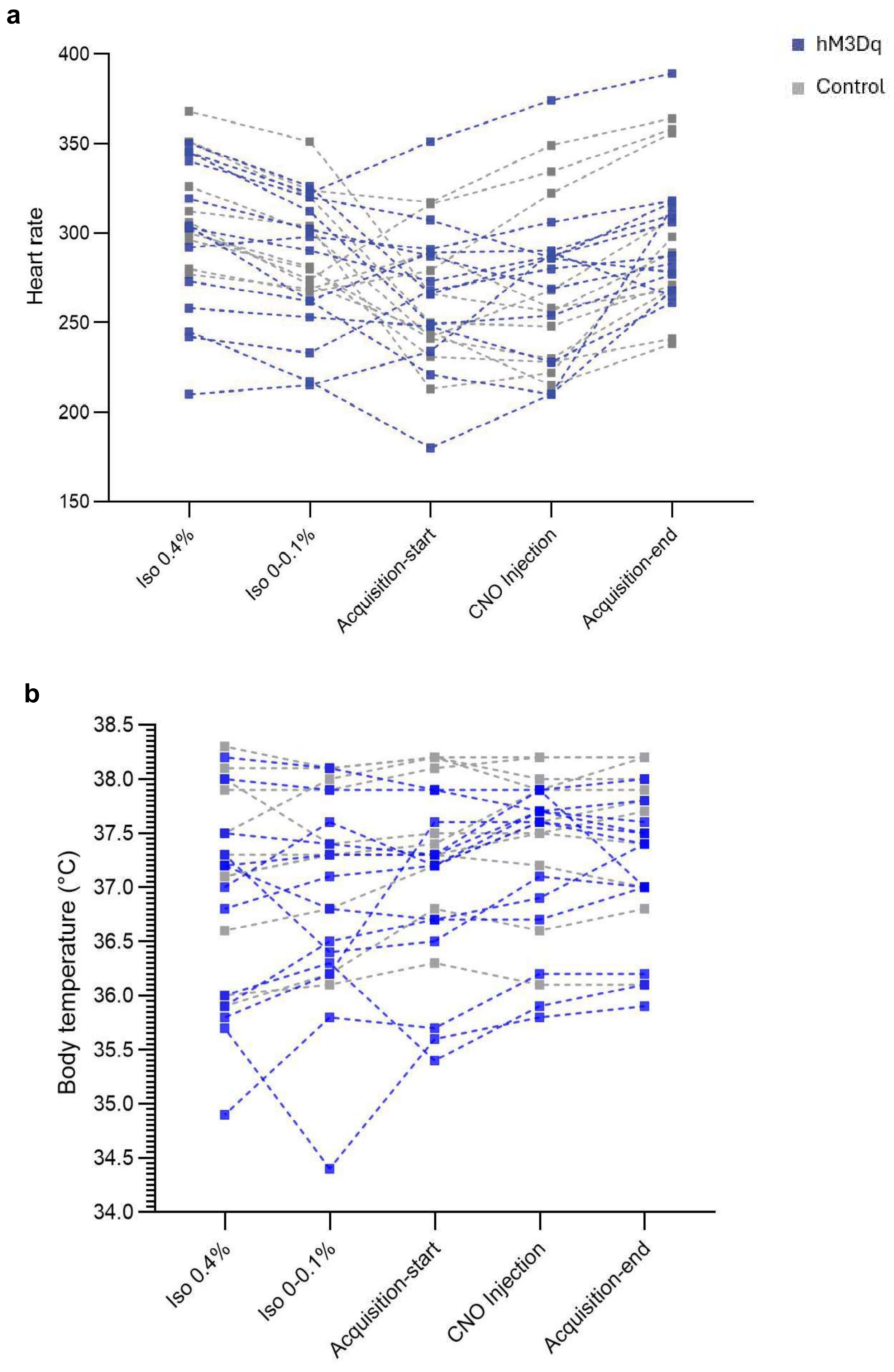

Figure S7. **Physiological parameters in fUS experiment. a**, heart rate. **b**, Body temperature.

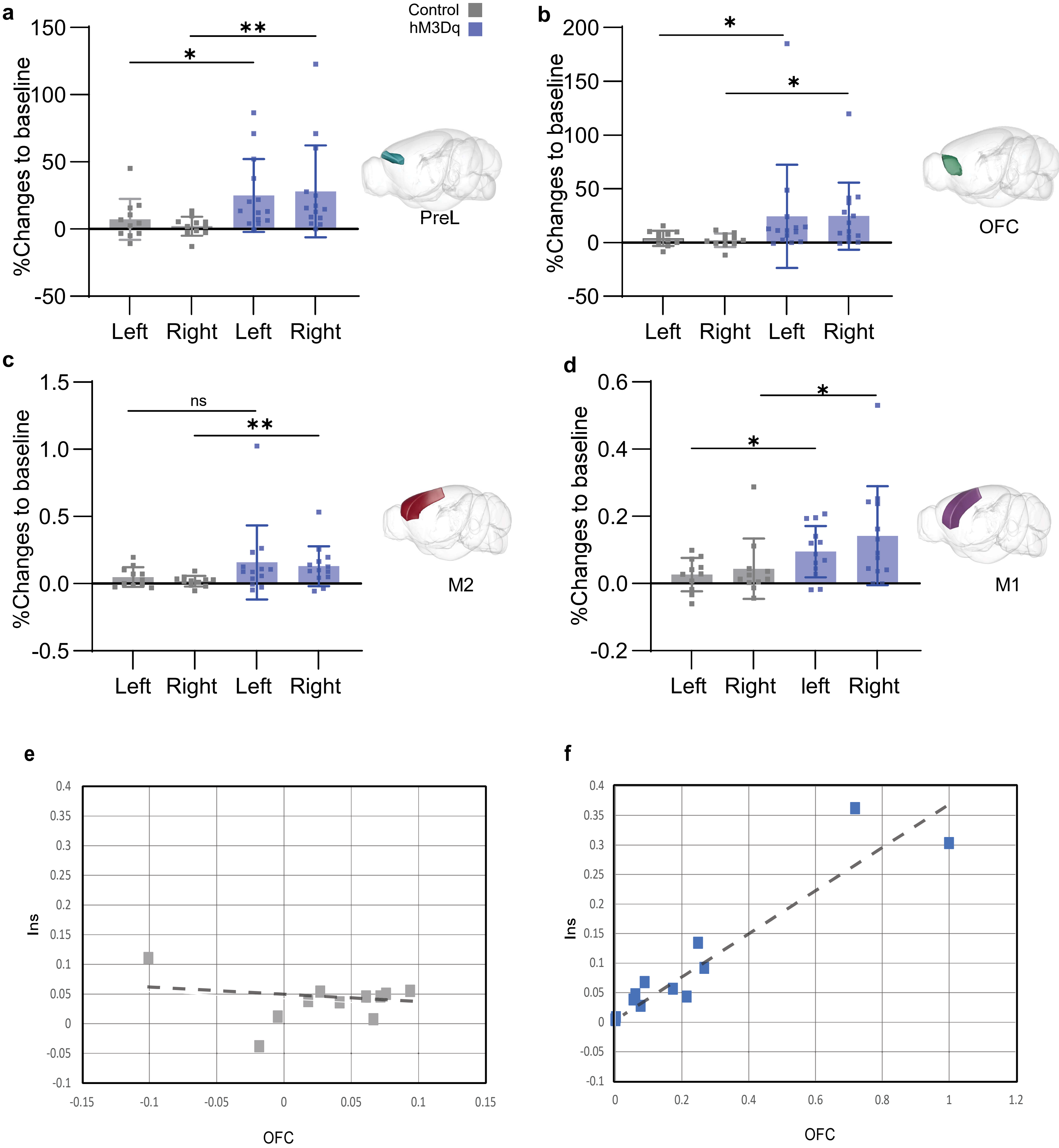
a
Control
hM3Dq
%Changes to baseline
150
100
50
0
-50
*
**
Left
Right
Left
Right
PreL
b
200
150
100
50
0
-50
*
*
%Changes to baseline
Left
Right
Left
Right
OFC
c
1.5
1.0
0.5
0.0
-0.5
ns
**
%Changes to baseline
Left
Right
Left
Right
M2
d
0.6
0.4
0.2
0.0
-0.2
*
*
%Changes to baseline
Left
Right
left
Right
M1
e
Ins
OFC
f
Ins
OFC

Figure S8. **CBV alterations following activation of PV interneurons in 4 regions. a,** Comparison of hemispheric rCBV values in PreL between control and hM3Dq groups after CNO application (Mann-Whitney test, *$p<0.05$, **$p<0.01$). **b**, Comparison of hemispheric rCBV values in OFC between control and hM3Dq groups after CNO application (Mann-Whitney test, *$p<0.05$). **c**, Comparison of hemispheric rCBV values in M2 between control and hM3Dq groups after CNO application (Mann-Whitney test, **$p<0.01$). **d**, Comparison of hemispheric rCBV values in M1 between control and hM3Dq groups after CNO application (Mann-Whitney test, *$p<0.05$). **e**, Correlation coefficient using Pearson's correlation between OFC and Insula in the control group ($r= -0,1882$). The significance of the correlation was tested at a 95% confidence level ($p= 0,5794$). **f**, Correlation coefficient using Pearson's correlation between OFC and Insula in the hM3Dq group ($r=0.9253$). The significance of the correlation was tested at a 95% confidence level ($p <0,0001$). Error bars represent mean ± standard deviation (SD).

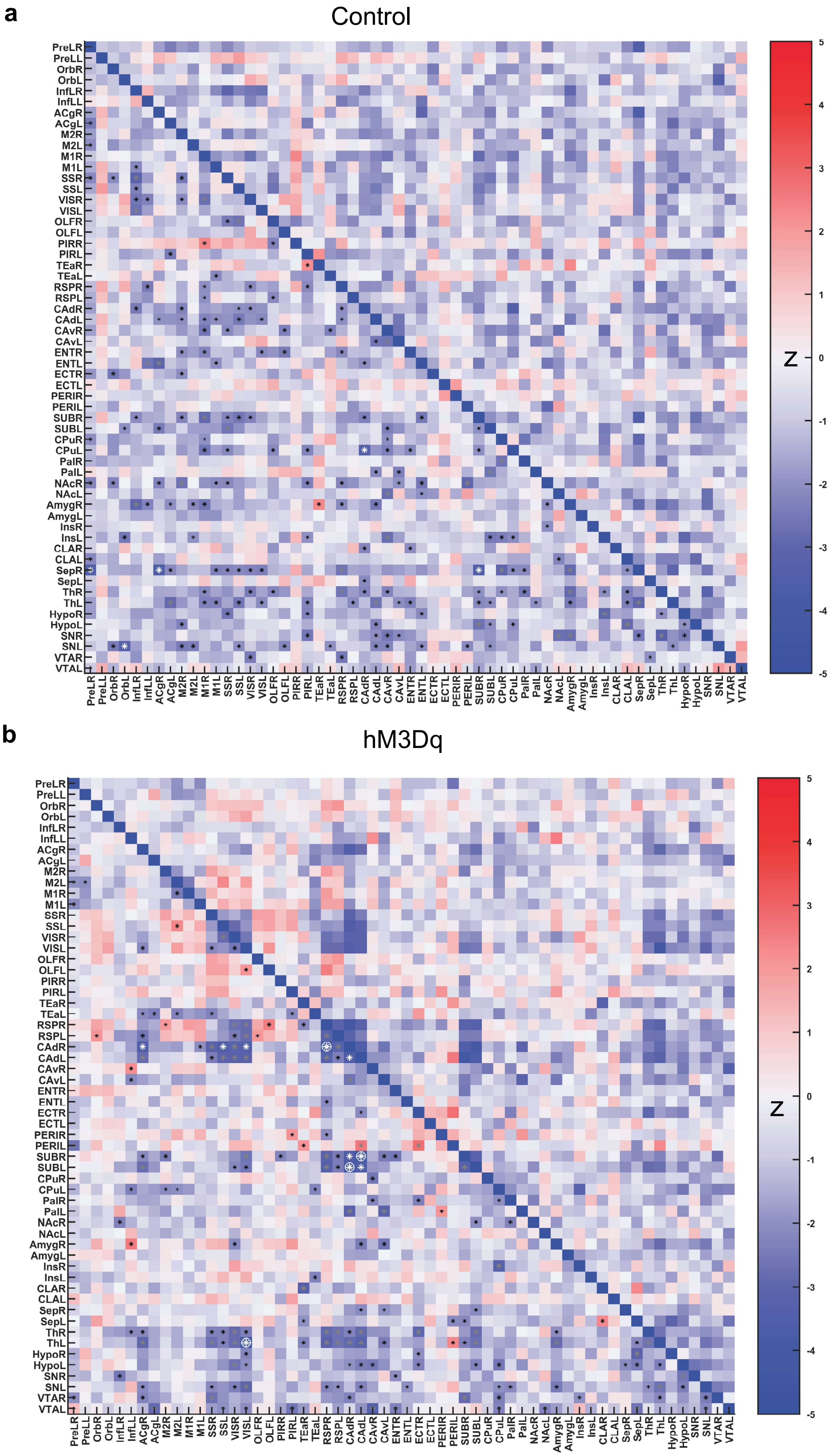

Figure S9. **FC alterations in whole brain regions following activation of PV interneurons in OFC.** The FC matrices demonstrate the Z-scores calculated between the readouts of the baseline and of the post CNO injection at the corresponding timepoints in 29 different regions (left and right hemisphere) in (**a**) control and (**b**) hM3Dq group (paired T test, black *p<0.05, gray*p<0.01, white *p<0.001, circle= FDR-corrected).

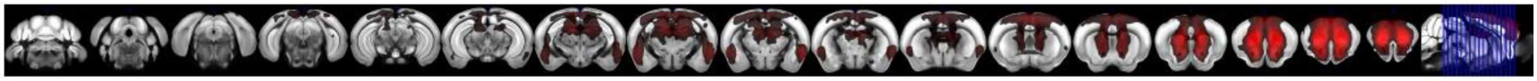

Figure S10, **OFC seed-to-voxel functional connectivity in male C57BL/6 mice with same sedation condition.**

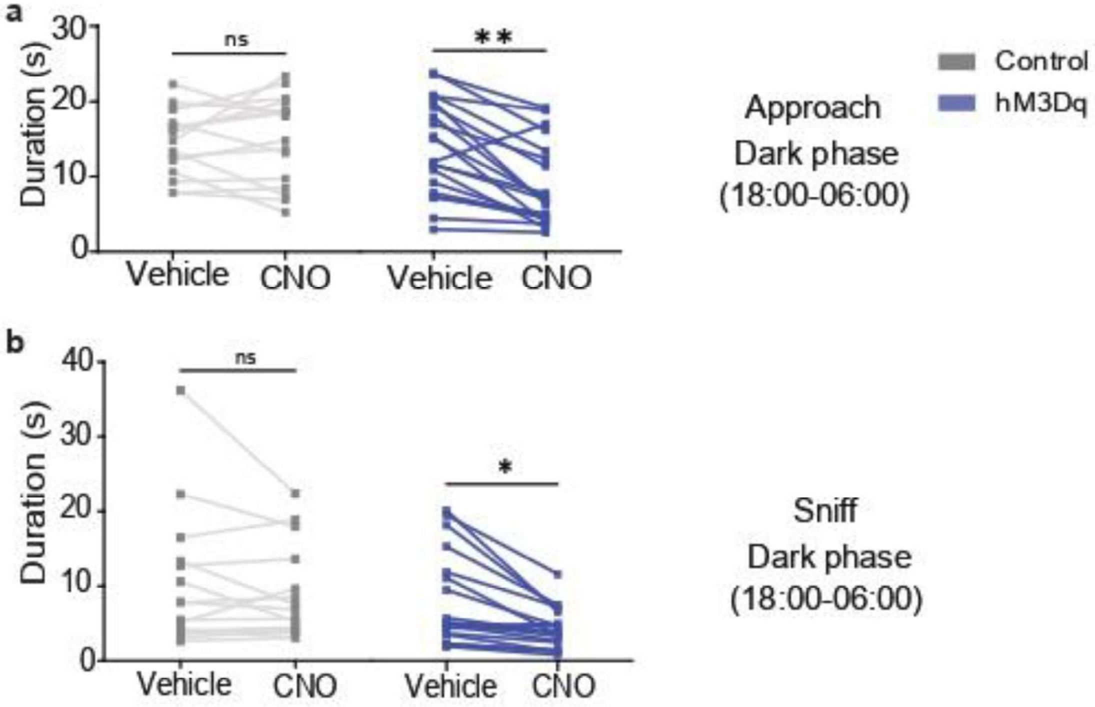

Figure S11, **Social impairment induced by OFC PV interneuron activation**. **a**, Social approach duration (average per hour) of individual animals in the control group (n=16) and in the hM3Dq group (n=20) during the 12 hours of the dark cycle after NaCl vehicle versus CNO application (unpaired T test, **p<0.01). **b**, Social sniff duration of individual animals in the control group (n=16) and in the hM3Dq group (n=20) during the 12 hours of dark cycle after NaCl versus CNO application (unpaired T test, *p<0.05).

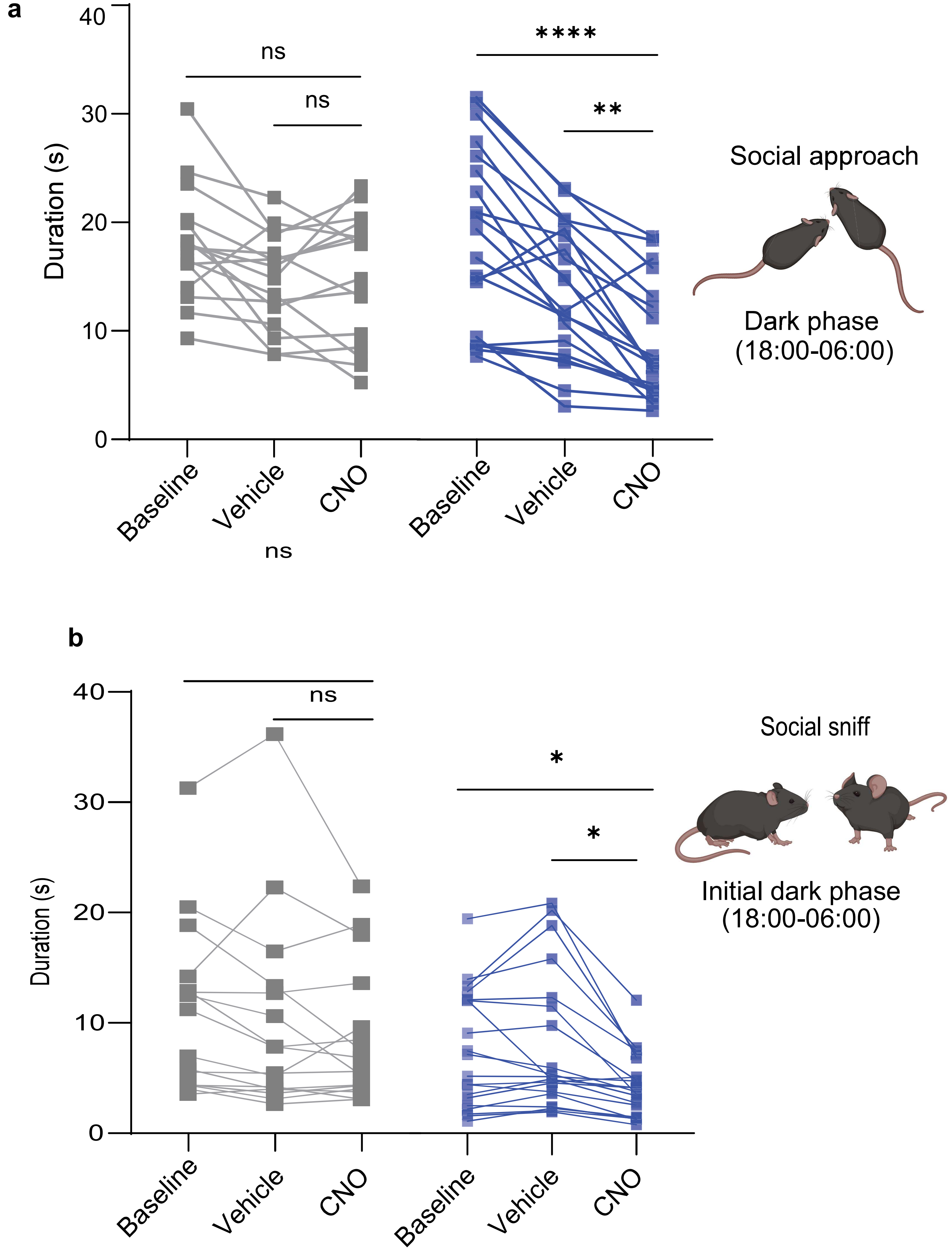
a
Duration (s)
40
30
20
10
0
ns
ns
****
**
Baseline
Vehicle
CNO
Baseline
Vehicle
CNO
ns
Social approach
Dark phase
(18:00-06:00)
b
Duration (s)
ns
*
*
Baseline
Vehicle
CNO
Baseline
Vehicle
CNO
Social sniff
Initial dark phase
(18:00-06:00)

Figure S12. **Comparing social approach and social sniff duration in baseline, vehicle and CNO days. a**, Social approach duration (average per hour) of individual animals in the control group (n=16) and in the hM3Dq group (n=20) during the 12 hours of the dark cycle (unpaired T test, ***$p<0.001$, **$p<0.01$). **b,** Social sniff duration (average per hour) of individual animals in the control group (n=16) and in the hM3Dq group (n=20) during the 12 hours of the dark cycle (unpaired T test, ***$p<0.001$, **$p<0.01$)

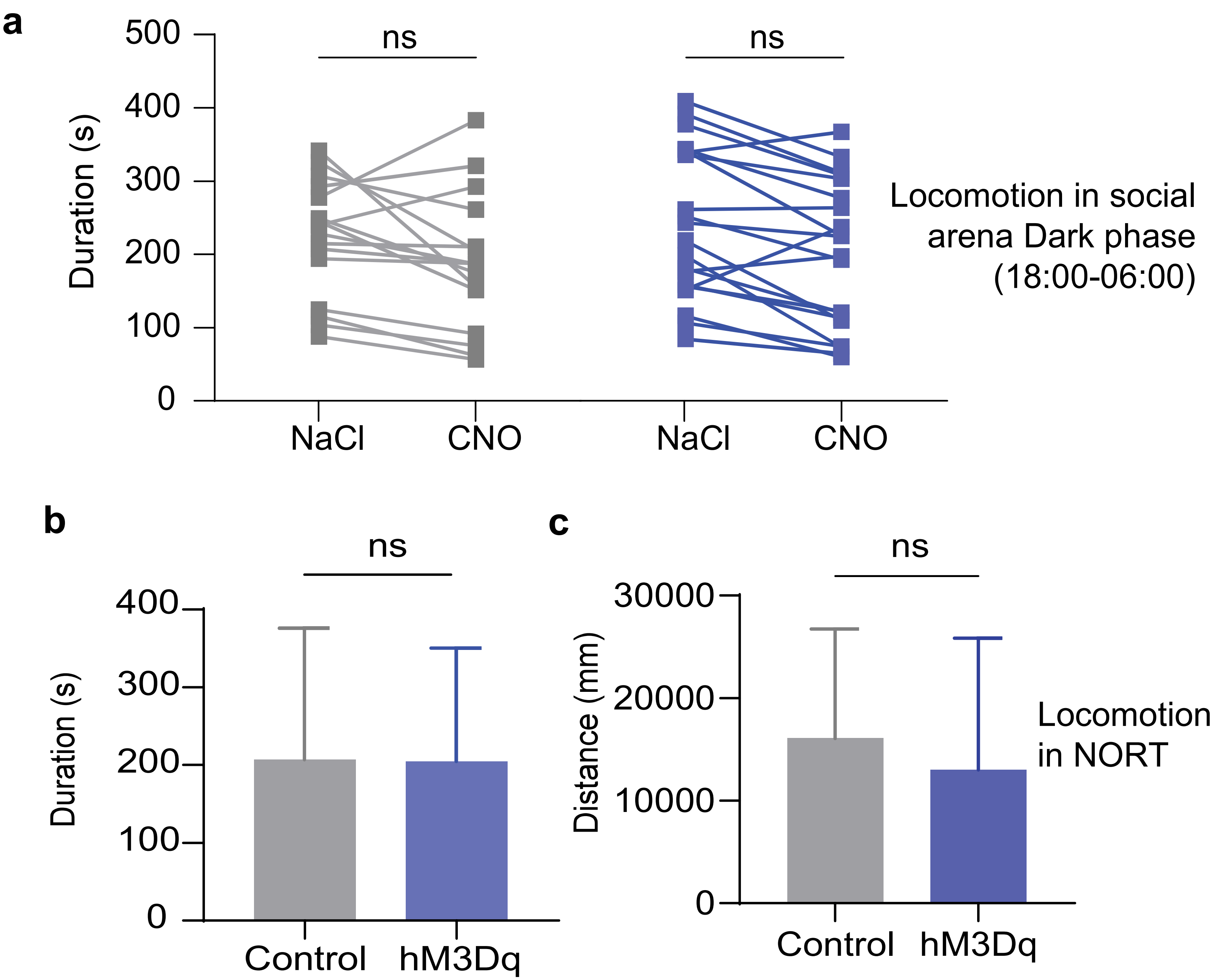

FigureS13. **Locomotion activity is not affected by PV interneurons modulation in OFC. a**, locomotion duration (seconds, average per mouse per hour) in the social arena during 12 hours dark phase in both control and hM3Dq groups. b, locomotion duration (seconds) each mouse as individual data points, in novel object recognition test (NORT) and **c**, distance, each mouse as individual data points, traveled in NORT experiment, shows significant difference between control and modulated groups. Error bars represent mean ± standard deviation (SD).

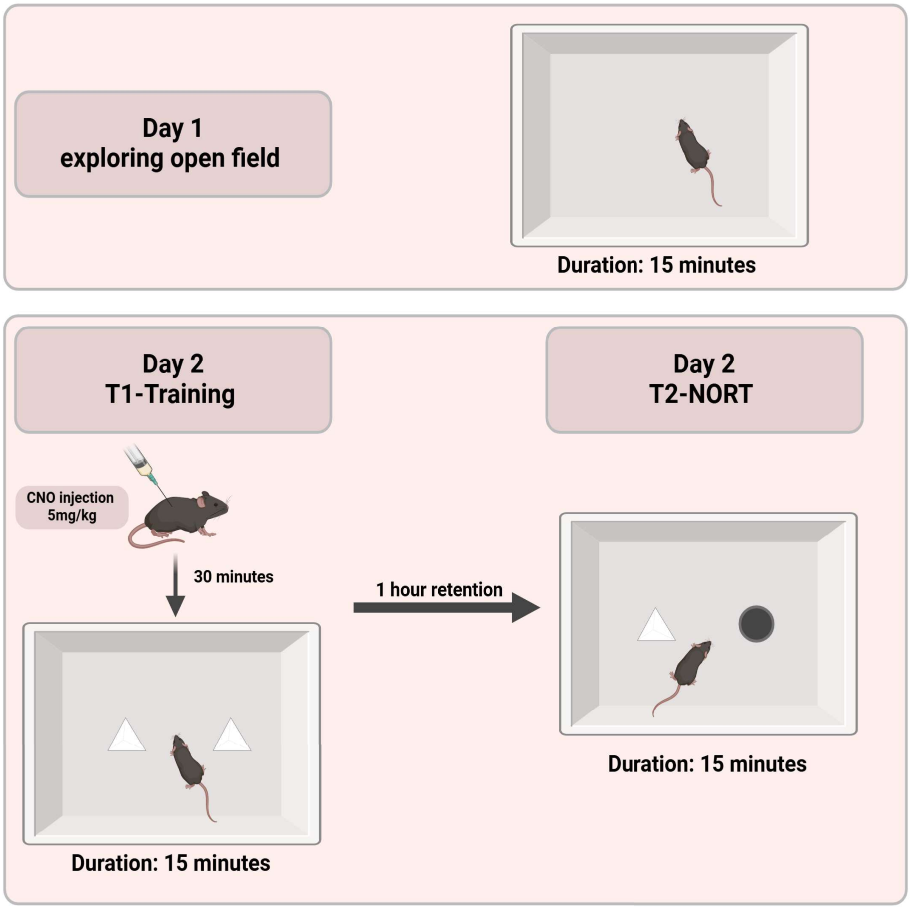

Figure S14. **NORT experimen**t conducted in 2 days in total, day one for exploration time and day two for training days. For the training phase (T1), the arena contained two identical objects, similar in shape (cone-shaped or triangular shape with a pointed tip) and color (black or white), positioned at the center. To investigate the influence of activating PV interneurons in the OFC on memory formation, the mice were injected with CNO 30 minutes before T1. For the novel object recognition test (T2), the mice were given a 1-hour retention period before being placed back in the arena for the third time. This time, one familiar object was replaced with a novel object that differed in color and shape.

Number of families	1
Number of comparisons per family	3
Alpha	0,05

Dunnett's T3 multiple comparisons test	Mean Diff,	95,00% CI of diff,	Below threshold?	Summary	Adjusted P Value	
Vehicle vs. Control CNO	-0,3212	-5,596 to 4,953	No	ns	0,9980	A-B
Vehicle vs. hM3Dq CNO	5,743	2,509 to 8,977	Yes	***	0,0002	A-C
Control CNO vs. hM3Dq CNO	6,064	0,7674 to 11,36	Yes	*	0,0218	B-C

Test details	Mean 1	Mean 2	Mean Diff,	SE of diff,	n1	n2	t	DF
Vehicle vs. Control CNO	12,38	12,70	-0,3212	2,056	30	16	0,1563	22,76
Vehicle vs. hM3Dq CNO	12,38	6,639	5,743	1,307	30	20	4,394	45,83
Control CNO vs. hM3Dq CNO	12,70	6,639	6,064	2,058	16	20	2,947	22,45

Compact letter display	
Control CNO	A
Vehicle	A
hM3Dq CNO	B

Table S1. **Results of Dunnett's T3 multiple comparisons test for approach.** evaluating differences among the Vehicle, Control + CNO, and hM3Dq + CNO groups.

Number of families	1
Number of comparisons per family	3
Alpha	0,05

Dunnett's T3 multiple comparisons test	Mean Diff,	95,00% CI of diff,	Below threshold?	Summary	Adjusted P Value	
Vehicle vs. Control CNO	1,283	-2,983 to 5,549	No	ns	0,8364	A-B
Vehicle vs. hM3Dq CNO	4,993	1,956 to 8,031	Yes	***	0,0006	A-C
Control CNO vs. hM3Dq CNO	3,710	0,2117 to 7,208	Yes	*	0,0359	B-C

Test details	Mean 1	Mean 2	Mean Diff,	SE of diff,	n1	n2	t	DF
Vehicle vs. Control CNO	7,936	6,653	1,283	1,711	36	16	0,7499	38,44
Vehicle vs. hM3Dq CNO	7,936	2,943	4,993	1,226	36	20	4,074	44,08
Control CNO vs. hM3Dq CNO	6,653	2,943	3,710	1,343	16	20	2,763	18,50

Compact letter display	
Vehicle	A
Control CNO	A
hM3Dq CNO	B

Table S2. **Results of Dunnett's T3 multiple comparisons test for sniff.** evaluating differences among the Vehicle, Control + CNO, and hM3Dq + CNO groups.
