# Supplementary material for: Orbitofrontal PV interneurons modulate social interaction via default mode network dynamics
Source: Commun Biol. 2026 Apr 24;9:573. doi: 10.1038/s42003-026-10060-y (PMC13109428; doi:10.1038/s42003-026-10060-y)
Supplement: Supplementary file 2 — Reporting Summary [file 42003_2026_10060_MOESM2_ESM.pdf]

nature portfolio

Corresponding author(s): Bastian Hengerer

Last updated by author(s): Oct 7, 2025

Reporting Summary

Nature Portfolio wishes to improve the reproducibility of the work that we publish. This form provides structure for consistency and transparency in reporting. For further information on Nature Portfolio policies, see our Editorial Policies and the Editorial Policy Checklist.

Statistics

For all statistical analyses, confirm that the following items are present in the figure legend, table legend, main text, or Methods section.

n/a	Confirmed	
☐	☒	The exact sample size (*n*) for each experimental group/condition, given as a discrete number and unit of measurement
☐	☒	A statement on whether measurements were taken from distinct samples or whether the same sample was measured repeatedly
☐	☒	The statistical test(s) used AND whether they are one- or two-sided<br>*Only common tests should be described solely by name; describe more complex techniques in the Methods section.*
☒	☐	A description of all covariates tested
☒	☐	A description of any assumptions or corrections, such as tests of normality and adjustment for multiple comparisons
☒	☐	A full description of the statistical parameters including central tendency (e.g. means) or other basic estimates (e.g. regression coefficient) AND variation (e.g. standard deviation) or associated estimates of uncertainty (e.g. confidence intervals)
☐	☒	For null hypothesis testing, the test statistic (e.g. *F*, *t*, *r*) with confidence intervals, effect sizes, degrees of freedom and *P* value noted<br>*Give P values as exact values whenever suitable.*
☒	☐	For Bayesian analysis, information on the choice of priors and Markov chain Monte Carlo settings
☒	☐	For hierarchical and complex designs, identification of the appropriate level for tests and full reporting of outcomes
☐	☒	Estimates of effect sizes (e.g. Cohen's *d*, Pearson's *r*), indicating how they were calculated

Our web collection on statistics for biologists contains articles on many of the points above.

Software and code

Policy information about availability of computer code

Data collection	Topscan- Ico scan
Data analysis	Topscan (cleversys Inc). Ico studio (Iconeus)-- SPM12-MRIcron-Matlab 2019

For manuscripts utilizing custom algorithms or software that are central to the research but not yet described in published literature, software must be made available to editors and reviewers. We strongly encourage code deposition in a community repository (e.g. GitHub). See the Nature Portfolio guidelines for submitting code & software for further information.

Data

Policy information about availability of data

All manuscripts must include a data availability statement. This statement should provide the following information, where applicable:

- Accession codes, unique identifiers, or web links for publicly available datasets
- A description of any restrictions on data availability
- For clinical datasets or third party data, please ensure that the statement adheres to our policy

The data supporting the findings of this study are available from the authors upon reasonable request.

Research involving human participants, their data, or biological material

Policy information about studies with human participants or human data. See also policy information about sex, gender (identity/presentation), and sexual orientation and race, ethnicity and racism.

Reporting on sex and gender	n/a
Reporting on race, ethnicity, or other socially relevant groupings	n/a
Population characteristics	n/a
Recruitment	n/a
Ethics oversight	n/a

Note that full information on the approval of the study protocol must also be provided in the manuscript.

Field-specific reporting

Please select the one below that is the best fit for your research. If you are not sure, read the appropriate sections before making your selection.

☒ Life sciences ☐ Behavioural & social sciences ☐ Ecological, evolutionary & environmental sciences

For a reference copy of the document with all sections, see nature.com/documents/nr-reporting-summary-flat.pdf

Life sciences study design

All studies must disclose on these points even when the disclosure is negative.

Sample size	Behavioral study: Cohort One: six weeks old B6;129P2-Pvalb<tm1(cre)Arbr>/J male mice (N = 36; weight range: 18-22 g)<br>NORT study: 20 mice from behavioral study<br>fUS study: Cohort two: six weeks old B6;129P2-Pvalb<tm1(cre)Arbr>/J male mice (N = 25; weight range: 18-22 g)
Data exclusions	strong fluctuations in body temperature during a scan
Replication	large sample size in behavioral study ands fUS study- behavioral results were replicated in another trials as well as c-fos expression. fUS data was also replicated in another trials but the data in not included due to age/Anesthesia protocol differences.
Randomization	all animals were randomly assigned to their groups from the same sex and age
Blinding	All data analysis was conducted in a blinded manner using specialized software. For the manual analysis of the NORT experiment, the scorers were also blinded to ensure objectivity.

Reporting for specific materials, systems and methods

We require information from authors about some types of materials, experimental systems and methods used in many studies. Here, indicate whether each material, system or method listed is relevant to your study. If you are not sure if a list item applies to your research, read the appropriate section before selecting a response.

Materials & experimental systems

n/a	Involved in the study	
☐	☒	Antibodies
☒	☐	Eukaryotic cell lines
☒	☐	Palaeontology and archaeology
☐	☒	Animals and other organisms
☒	☐	Clinical data
☒	☐	Dual use research of concern
☒	☐	Plants

Methods

n/a	Involved in the study	
☒	☐	ChIP-seq
☒	☐	Flow cytometry
☒	☐	MRI-based neuroimaging

Antibodies

Antibodies used	Primary antibodies:

Antibodies used

1.rabbit polyclonal anti c-Fos (abcam, GB, #ab190289, 1:1000)
https://www.abcam.com/en-us/products/primary-antibodies/c-fos-antibody-bsa-free-ab190289#tab=datasheet

2.guinea pig anti-parvalbumin (Synaptic Systems, DE, #195004, 1:500)
https://www.sysy.com/product/195004

secondary antibodies:
1. anti-rabbit Alexa Fluor 488 (Invitrogen, #A11070, 1:1000) https://www.thermofisher.com/antibody/product/Goat-anti-Rabbit-IgG-H-L-Cross-Adsorbed-Secondary-Antibody-Polyclonal/A-11070
2. goat anti-guinea pig Alexa Fluor 647 (ThermoFisher, US, #A21450, 1:1000)

Validation

Primary antibodies:
1.manufacturer statement: Anti-c-Fos antibody - BSA free (ab190289) was first used in a scientific publication in 2015 and has been cited over 216 times in peer reviewed journals. It's performance in IHC in mouse and rat samples is trusted by the scientific community.
Abcam's high quality validation processes ensure Anti-c-Fos antibody - BSA free (ab190289) has high sensitivity and specificity.
2.#195004: https://pubmed.ncbi.nlm.nih.gov/39300067/

Secondary antibodies:
1.Anti-Rabbit secondary antibodies are affinity-purified antibodies with well-characterized specificity for rabbit immunoglobulins and are useful in the detection, sorting or purification of its specified target. Secondary antibodies offer increased versatility enabling users to use many detection systems (e.g. HRP, AP, fluorescence). They can also provide greater sensitivity through signal amplification as multiple secondary antibodies can bind to a single primary antibody. Most commonly, secondary antibodies are generated by immunizing the host animal with a pooled population of immunoglobulins from the target species and can be further purified and modified (i.e. immunoaffinity chromatography, antibody fragmentation, label conjugation, etc.) to generate highly specific reagents.
Target Information
2.Anti-Guinea Pig secondary antibodies are affinity-purified antibodies with well-characterized specificity for guinea pig immunoglobulins and are useful in the detection, sorting or purification of its specified target. Secondary antibodies offer increased versatility enabling users to use many detection systems (e.g. HRP, AP, fluorescence). They can also provide greater sensitivity through signal amplification as multiple secondary antibodies can bind to a single primary antibody. Most commonly, secondary antibodies are generated by immunizing the host animal with a pooled population of immunoglobulins from the target species and can be further purified and modified (i.e. immunoaffinity chromatography, antibody fragmentation, label conjugation, etc.) to generate highly specific reagents.

Animals and other research organisms

Policy information about studies involving animals; ARRIVE guidelines recommended for reporting animal research, and Sex and Gender in Research

Laboratory animals

six weeks old B6;129P2-Pvalb<tm1(cre)Arbr>/J male mice. they were obtained from Charles Rivers Research Models and Services (Germany). Mice were kept under standard conditions (12h/12h dark/light cycle, temperature 20-24 °C, humidity 45-65%, and ad libitum access to food and water) in groups of four. After a two-week acclimation period, stereotaxic surgery was performed.

Wild animals

n/a

Reporting on sex

finding applies on male sex. no female experiment was conducted.

Field-collected samples

n/a

Ethics oversight

All experiments were approved by the responsible governmental animal ethics committee (Regierungspräsidium Tübingen, Baden-Württemberg, Germany).

Note that full information on the approval of the study protocol must also be provided in the manuscript.

Plants

Seed stocks

n/a

Novel plant genotypes

n/a

Authentication

n/a
